# KIR Genes and Patterns Given by the A Priori Algorithm: Immunity for Haematological Malignancies

**DOI:** 10.1155/2015/141363

**Published:** 2015-09-30

**Authors:** J. Gilberto Rodríguez-Escobedo, Christian A. García-Sepúlveda, Juan C. Cuevas-Tello

**Affiliations:** ^1^Facultad de Ingeniería, Universidad Autónoma de San Luis Potosí, Avenida Dr. Manuel Nava No. 8, Zona Universitaria, 78290 San Luis Potosí, ZC, Mexico; ^2^Laboratorio de Genómica Viral y Humana, Facultad de Medicina, Universidad Autónoma de San Luis Potosí, Avenida Venustiano Carranza No. 2405, Colonia Filtros las Lomas, 78210 San Luis Potosí, CP, Mexico

## Abstract

Killer-cell immunoglobulin-like receptors (KIRs) are membrane proteins expressed by cells of innate and adaptive immunity. The KIR system consists of 17 genes and 614 alleles arranged into different haplotypes. KIR genes modulate susceptibility to haematological malignancies, viral infections, and autoimmune diseases. Molecular epidemiology studies rely on traditional statistical methods to identify associations between KIR genes and disease. We have previously described our results by applying support vector machines to identify associations between KIR genes and disease. However, rules specifying which haplotypes are associated with greater susceptibility to malignancies are lacking. Here we present the results of our investigation into the rules governing haematological malignancy susceptibility. We have studied the different haplotypic combinations of 17 KIR genes in 300 healthy individuals and 43 patients with haematological malignancies (25 with leukaemia and 18 with lymphomas). We compare two machine learning algorithms against traditional statistical analysis and show that the “a priori” algorithm is capable of discovering patterns unrevealed by previous algorithms and statistical approaches.

## 1. Introduction

One goal in systems biology, along with functional genomic (Human Genome Project) analysis and physiology (Human Physiome Project), is to provide personalized medicine in a practical, clinically useful way. The digital genome and environmental signals are two fundamental types of biological information that dictate whether an individual adopts a normal or diseased phenotype. Therefore, functional genomics data can help diagnose disease and guide therapy [1].

Several cancer research initiatives employing genomic information focus mainly on DNA microarray data in the search for biomarkers using tens of thousands of genetic polymorphisms [2]. However, after recent discoveries relating to KRAS gene mutations in cancer patients, novel research strategies are focusing on circulating tumour DNA (ctDNA) and to the way that it might allow for a closer surveillance of the clinical evolution of cancer in certain types of patients [3]. Several diseases have been studied in systems biology; this paper focuses on haematological malignancies (leukaemia and lymphomas). Contrary to DNA microarray data and ctDNA, this paper studies the impact of specific innate immunity genes with disease occurrence or protection.

Traditionally, hypothesis driven approaches based on current knowledge have been used to uncover associations between a small number of genetic traits and disease occurrence or disease progression. Genome-wide analysis studies (GWAS) have rapidly become powerful tools for the analysis of tens of thousands and sometimes millions of genetic markers and of their association with complex diseases. In the last 15 years, several GWAS have demonstrated the importance that immune and nonimmune gene polymorphisms have at determining an individual's capability to mount an immune response against infectious pathogens, residual leukaemia, antileukaemia drug metabolism, and haemopoietic stem cell transplantation (HSCT) outcome. However, only a few studies have addressed the importance of analysing the full context of innate immunity genes and of their interplay with the adaptive immune system with regards to leukaemias and lymphomas. In more recent years network-assisted analysis (NAA) of GWAS data has demonstrated enormous power for the study of various human diseases or traits [4–7].

A small subset of CD8 lymphocytes and Natural Killer (NK) cells are represented by the Killer-Cell Immunoglobulin-like receptors (KIR), and they are key participants of immune responses to tumours. KIR genes, in comparison to genes of the adaptive immune system, are genetically predetermined and remain unchanged throughout life [8, 9]. Nowadays, 17 KIR genes have been discovered, which exhibit allelic polymorphism [10], forming a cluster in the locus 19q13.4. The KIR genes are physically contiguous strings, known as haplotypes [11, 12]. The variability in KIR genotype is such that most pairs of unrelated human individuals have different KIR genotypes, so the unique feature of the human KIR system is the representation of two distinctive groups of haplotypes (A and B), and many haplotypes having presence and absence of genes and variants are known [13]. A KIR haplotype is composed of two motifs, centromeric and telomeric. The KIR haplotype motifs are cA01, cB01, cB02, cB03, tA01, and tB01 [11, 12]. The KIR haplotypes of the great majority of individuals contain the four framework genes KIR3DL3, KIR3DP1, KIR2DL4, and KIR3DL2 [11, 14].

KIR genes encode for two (2D) or three (3D) extracellular domain membrane bound proteins capable of transducing activating (S) or inhibitory (L) signals on binding of their cognate ligands. It is the balance and integration of these signals that modulates NK cell cytotoxicity and cytokine release. The haplotypes of group A are more important because they have simple and constant gene content, dominated by inhibitory genes (L). On the other hand, haplotypes of group B have variable and greater gene content, involving both inhibitory and activating receptors [11]. NK cells were initially identified by their ability to spontaneously kill tumour cells without prior sensitisation [15–17]. Historical studies of the immunogenetic factors that determine clinical outcome in patients subjected to HSCT for haematological malignancies were the first to highlight the clinical relevance of KIR genes in antitumour responses [18].

The first study to suggest such an association described a potent graft-versus-leukaemia effect arising from predicted NK cell alloreactivity in the Graft-versus-Host direction amongst patients subjected to HSCT for leukaemias [19]. Many other studies published since then have described KIR gene associations with antitumour effects and posttransplant clinical endpoints [20–26]. In addition, NK cell antitumour activity has been demonstrated* in vitro* against a wide variety of haematological malignancies [18, 27]. In all, these findings support the notion that KIRs allow NK cells to play an important role at determining susceptibility to certain haematological tumours [28–30].

Previous findings based on our data employing multivariate analysis of KIR carrier frequencies with a traditional statistical comparison (contingency tables using Pearson's or Fishers' exact test [31]) revealed only that KIR2DL2 was more frequent amongst patients with haematological malignancy in comparison to the healthy donors (*p* ≤ 0.0001). Decision trees (ID3 algorithm [32]) generated at 50% and 75% training data also provided support the importance of KIR2DL2 [33]. Other findings produced with the ID3 algorithm on our similar data suggest a protective effect for (i) cB03 motif (KIR2DL3, KIR2DL5, KIR2DS5, KIR2DP1, and KIR2DL1 genes) in agreement with KIR3DS1-2DL5-2DS5-2DS1 genotype with protection from Hodgkin's lymphoma [34]; (ii) KIR3DS1 gene (only provided a protective effect when observed in the absence of KIR2DL2 or KIR2DL5 genes) as suggested previously [25, 34, 35]; and (iii) KIR2DS1 when present together with KIR2DL2, KIR2DS2, and KIR2DL3 but in the absence of KIR3DL1 [33].

Nevertheless, the ID3 algorithm failed to find associations related to the KIR2DS3, as described previously by others researchers [35–37]. Neither KIR2DL1 nor KIR2DL3 are on their own important factors in the ID3 decision processes [33]. One reason is that the ID3 algorithm is based only on entropy of information, which could not identify other patterns with this measure of information. Genes KIR2DL1, KIR2DL3, KIR2DL5, and KIR2DS3/S5 were also present in our patients in haplotype motifs other than the classic cA01 (or KIR2DL1 and -2DL3) and cB01 (for the KIR2DL1, KIR2DL5, KIR2DS3, and KIR2DS5), as suggested for certain Hodgkin's lymphomas [38]. Differences in patient demographics, clinical management, KIR typing method, and the preferred transplant modality have largely contributed to the heterogeneity of the KIR gene associations that have been described across the literature.

In this paper, we further study the* a priori* algorithm on the same dataset in an effort to discover novel associations not identified by the ID3 algorithm. The* a priori* algorithm is an algorithm that belongs to the family of data mining algorithms in the field of machine learning and artificial intelligence [39–41]. Regarding classification algorithms, previous research has already described the potential that support vector machines (SVM) have [33], as well as that of other state-of-the-art classification algorithms including Deep Neural Networks and Convolutional Neural Networks [42]. Moreover, research on classification algorithms is also focusing on creating an ensemble of classifiers such as LibD3C [43]. However, these algorithms are deficient at finding association rules and defining them, so more research is needed. As our work with KIR and haematological malignancies represents an imbalanced classification problem [44], the a priori algorithm was considered as an interesting and informative approach for work with this dataset. The main contributions of this paper are (i) we follow a data mining methodology to study associations between KIR genes and disease; (ii) the novel application of the a priori algorithm to identify associations between KIR genes and haematological malignancies; (iii) we found novel associations not detected before by the ID3 algorithm (see Section 3) (iv) we apply an improved version of the ID3 algorithm, known as J48, so one can validate that the results of the a priori algorithm are novel.

## 2. Materials and Methods

### 2.1. Study Population

Samples belonging to the Mexican Reference Genomic DNA Collection (MGDC-REF), which includes 300 unrelated blood donors, were used as healthy controls for this study. This Mexican mestizo reference population included 135 (45%) males and 165 (55%) females aged between 19 and 38 years (median of 24) of which 75% were residents of the city of San Luis Potosí and 25% were residents of rural areas of this Mexican state. These DNA samples were extracted from blood-bank discarded leukocyte concentrates referred to us by Hospital Central “Dr. Ignacio Morones Prieto” according to previously published protocols [45]. A more detailed description of the KIR features present in this reference population is given in the original publication [46]. In addition, 43 DNA samples obtained from patients with haematological malignancies (25 with leukaemia and 18 with lymphomas) referred to us by the Haematology Department of Hospital Central “Dr. Ignacio Morones Prieto” were included as representatives of a diseased study group. More information for the haematological cohort is given in Table 1. All samples were provided to us in accordance with state and national ethics regulations and lacking personal identifying information so as to ensure patient/donor confidentiality.

### 2.2. KIR Genotyping and Encoding

**Figure 1:**
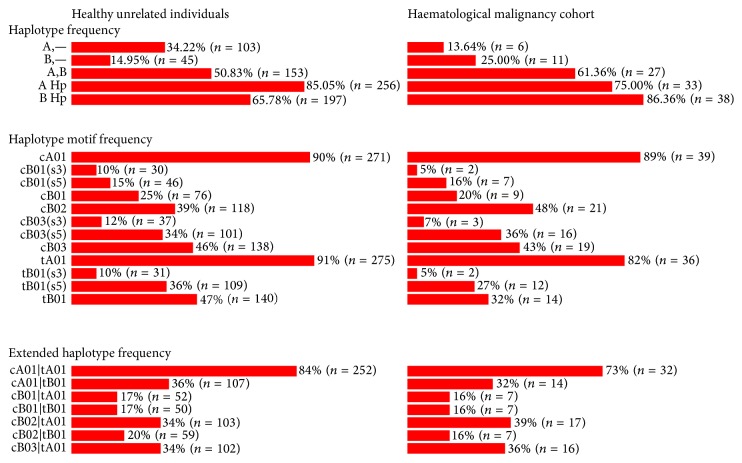
KIR gene features present in the healthy unrelated donor and haematological malignancy cohorts. KIR haplotype. A,—corresponds to group A homozygous haplotypes, whereas A Hp includes both homozygous and heterozygous group A haplotypes (vice versa for B). cB01 haplotypes having KIR2DS3 but not KIR2DS5 are indicated as “cB01(s3),” vice versa for those containing KIR2DS5 instead of KIR2DS3. The same applies to cB03 and tB01 categories. Combinations of centromeric and telomeric motifs that are thought to be very likely occurring based on Pyo's 2010 criteria [11] have been included at the bottom of the figure as extended haplotypes.

KIR gene content was determined using a locally developed sequence specific priming polymerase chain reaction (SSP-PCR) genotyping technique capable of detecting the presence or absence of each of the 17 genes [46]. This SSP-PCR approach did not enable us to distinguish between KIR2DL5A and KIR2DL5B nor the centromeric/telomeric localisation of genes. PCR amplicons were resolved in 1.5% agarose gels and digitally documented after ethidium bromide staining. Genotypes having KIR2DL2, KIR2DL5, KIR2DS1, KIR2DS2, KIR2DS3, KIR2DS5, or KIR3DS1 were considered to have at least one group B haplotype. Genotypes having KIR2DL3, KIR2DP1, KIR2DL1, KIR3DL1, and KIR2DS4 in the absence of any group B haplotype gene were classified as homozygous for group A haplotypes. Genotypes having all group A haplotype genes with at least one group B defining gene were considered heterozygous for groups A and B haplotypes. Centromeric and telomeric KIR haplotype motifs were deterministically inferred for the 300 samples after manually comparing their genotyping profile to that of the previously described KIR haplotype motifs based on criteria published previously by Pyo et al. [11]; see also Table 1 [46]. Similarly, KIR gene content haplotypes were inferred for the eleven most frequent genotypes observed in our population (present in >1% of our study population) based on Pyo's criteria [11]. As our genotyping approach does not resolve cis and trans relationships between genes, other haplotype motifs and/or haplotype combinations cannot be ruled out.  Figure 1 provides overall classical KIR haplotype, haplotype motif, and extended haplotype frequencies for both study cohorts as provided by our online tool KIRHAT (KIR gene Haplotype Analysis Tool (KIRHAT) available through http://www.genomica.uaslp.mx).

Since KIR haplotype motifs can be inferred from genotyping results as described with greater detail in the original publication [11] and with the fact that the KIR haplotypes of the great majority of individuals contain the four framework genes KIR3DL3, KIR3DP1, KIR2DL4, and KIR3DL2 [11, 14]. Then, we only focus on the following 12 KIR genes: KIR2DL1, KIR2DL2, KIR2DL3, KIR2DL5, KIR2DS1, KIR2DS2, KIR2DS3, KIR2DS4, KIR2DS5, KIR2DP1, KIR3DL1, and KIR3DS1.

KIR gene encoding strings included information for the 12 genes for each of the 343 samples, stored in rows; see Table 2. Additionally, we have included a health status variable (*C*, known as class), which was =1 in samples obtained from individuals having a haematological malignancy and 0 in healthy donors, as shown in the last column of Table 2.

### 2.3. Traditional Statistical Tests

KIR gene carrier frequencies were calculated by direct counting of the number of individuals bearing a genetic trait. KIR gene and haplotype carrier frequency comparisons between healthy controls and diseased patients employed a two-sided Pearson's *χ* ^2^ or Fisher's exact test, significance being established at *p* < 0.05. This test is also known as 2-way contingency table analysis [31].

### 2.4. J48 Algorithm

The ID3 algorithm was originally introduced by Quinlan in 1983, and it is used for automatic rule generation in expert systems [32]. ID3 is also employed as a data mining tool to generate decision trees by using information entropy. Improved versions of ID3 include C4.5 and C5 algorithms. The J48 algorithm belongs to this class of algorithms for generating C4.5 decision trees [47].

### 2.5. A Priori Algorithm

This algorithm is used to find association rules given a dataset [39, 48]. A rule has two main components: the* if* and* then* part and the antecedent and the consequent part, respectively. We are going to use the symbols ==> or ⇒ to separate those components of a rule. When several variables are involved within the* if* part, we consider the logical operator* and* (inclusive).

#### 2.5.1. A Toy Example for the A Priori Algorithm

Before a formal explanation of the algorithm is given, a toy example with two genes (variables) is given. Let us consider only two genes (*g*1, *g*2, 0 indicates absence of gene while 1 indicates presence) and the clinical outcome (class 0 for healthy subjects and 1 for diseased); see Table 3. One can clearly see that only the cooccurrence of both genes leads to a diseased phenotype in this example while other combinations of the genes lead to a normal phenotype. In this specific case, the underlying behavior is best described by the AND operator (∧), in logic, where the performance is given by a truth table; see Table 3.

Based on this simple example, we then proceed to create an artificial dataset; see Table 4.

**Figure 2:**
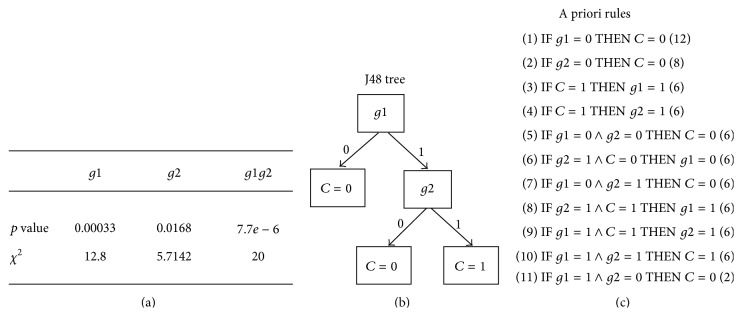
Results from the example. (a) Statistical test. (b) J48 pruned tree. (c) Rules given by the a priori algorithm.

The dataset in Table 4 simulates 20 individuals, with only two genes (*g*1 and *g*2), and one class (*C*). If we apply a statistical analysis, we obtain the statistically significant *p* values of cross-tabulation comparison (shown in Figure 2(a)). Likewise, by applying the J48 algorithm a pruned tree (given in Figure 2(b)) is generated detailing associations rules along with a summary of those rules generated by the a priori algorithm (given in Figure 2(c)).

From this example, we can observe the following.

*(1) Statistical Analysis.* Here we show both univariate and multivariate statistical analyses. The column *g*1*g*2 combines the two variables *g*1 and *g*2. Since we apply the AND operator for combining variables, the data of the column *g*1*g*2 and *C* (Class) are the same. Therefore, the smallest *p* value is for the combined variable *g*1*g*2. However all *p* values are lower than our threshold (*p* < 0.05), so the results for all variables are statistically significant (or correlated). This is all we can infer from this simple statistical analysis.

*(2) J48*. The decision tree generated by the J48 algorithm agrees with the statistical analysis; the most important variable is *g*1, because it is at the first level of the tree. Moreover, it tells us that if the variable *g*1 is 0, then variable *C* is also 0. Still, it tells us that if variable *g*1 is 1, then we need to look at variable *g*2 to decide the value for *C*.

*(3) A Priori Algorithm*. This algorithm gives us the total of rules that can be inferred from the dataset in Table 3, which is all possible combinations among variables including the class variable (*C*). Besides, it also gives the most important rules, the first ones; that is, *g*1 = 0  =>  *C* = 0. This rule agrees with the statistical analysis and the J48 decision tree. The number (12), that is, the frequency, within the first rule, indicates how many times this rule applies in the whole dataset. Moreover, we can ask the algorithm to mine for class association rules, as we are only interested in rules where the class (*C*) appears as the consequent part of the rule:

(1) IF *g*1 = 0 THEN *C* = 0 (12)
(2) IF *g*2 = 0 THEN *C* = 0 (8)
(3) IF *g*1 = 0  ∧  *g*2 = 0 THEN *C* = 0 (6)
(4) IF *g*1 = 0  ∧  *g*2 = 1 THEN *C* = 0 (6)
(5) IF *g*1 = 1  ∧  *g*2 = 1 THEN *C* = 1 (6)
(6) IF *g*1 = 1  ∧  *g*2 = 0 THEN *C* = 0 (2).

If one observes these 6 rules, apart from the two first rules, they show the full performance of the AND operator, as shown in the truth table; see Table 3. It also tells us that if *g*1 = 0 then *C* = 0, regardless of the value of *g*2, and the same happens when *g*2 = 0. Finally, this result captures the main rule, which establishes the only case when *C* = 1; that is, *g*1 = 1 and *g*2 = 1.

#### 2.5.2. Formal Definition of the A Priori Algorithm

Let us define formally the a priori algorithm, so *I* = {*i*_1_, *i*_2_, *i*_3_,…, *i*_*m*_} is a set of binary attributes called items. *D*⊆*ℙ*(*I*) is a set of transactions, where *ℙ* denotes the power set of *I*, that is, all subsets of *I*. For example, the power set of *S* = {*a*, *b*} is *ℙ*(*S*) = {{}, {*a*}, {*b*}, {*a*, *b*}, {*b*, *a*}}. We are looking for implications, rules, of the form *X*⇒*Y*, where *X*⊆*I*, *Y*⊆*I*, and *X*∩*Y* = *ϕ*. We measure the quality of the rule by the following: (i) the support is the number of transactions where the antecedent of the rule is present, that is, supp(*X*) = |*X*|/|*D*|; (ii) the confidence measures the strength of the rule, and this measure is based on the support, where confidence(*X*⇒*Y*) = supp(*X* ∪ *Y*)/supp(*X*) = |*X* ∪ *Y*|/|*X*|; (iii) the correlation of a rules is based on probabilities, where correlation(*X*⇒*Y*) = *P*(*X* ∪ *Y*)/*P*(*X*)*P*(*Y*) [39, 48].

The pseudocode of the a priori algorithm [39, 48] is shown in Pseudocode 1.

#### 2.5.3. Our Model

For our dataset of 12 KIR genes with the information of the 343 donors, as illustrated in Table 2, we use a set *I* = {*i*_1_, *i*_2_, *i*_3_,…, *i*_13_} with 13 items. The first twelve items represent the KIR genes, where *i*_*j*_ = 1 if the gene is present, and *i*_*j*_ = 0 if it is not. The item *i*_13_ corresponds to the class (*C*), where 0 indicates when the donor is healthy and 1 when the donor has some hematological malignancy (disease). The set *D* corresponds to the 343 donors; we are interested in association rules of the form

(1) $$\begin{array}{c} \left(\;i_{j}=v_{j}\;\right)\wedge \left(\;i_{k}=v_{k}\;\right)\wedge \cdots \wedge \left(\;i_{l}=v_{l}\;\right)⟹C, \end{array}$$

where *v*_*j*_, *v*_*k*_,…, *v*_*l*_ are the values of each item (0 or 1) and *C* denotes the class. Also the set {*i*_*j*_, *i*_*k*_, …, *i*_*l*_}⊆*I*, where *j* ≠ *k* ≠ ⋯≠*l*.

### 2.6. Weka

**Figure 3:**
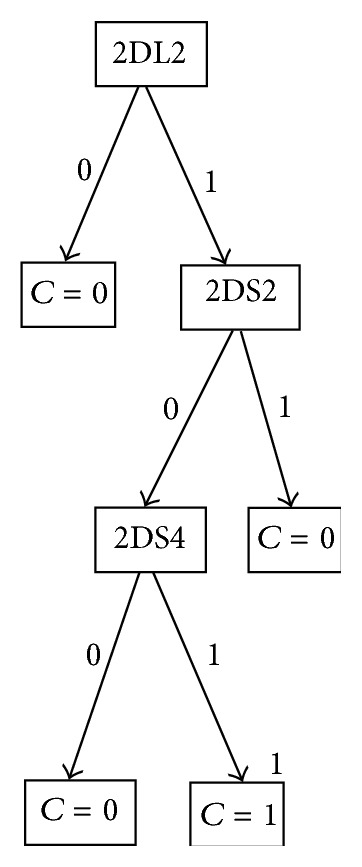
J48 decision tree.

The software that we use for our experiments is called Weka (http://www.cs.waikato.ac.nz/~ml/weka/index.html) [49]. It is open source software under the GNU general public license. The motivation of this software project is the invention and application of machine learning methods, so computer programs can automatically analyze large datasets. The results of these machine learning algorithms, in particular data mining algorithms, can be used to automatically make predictions or help people make decisions faster and accurately [49]. Weka contains a collection of machine learning algorithms for data mining tasks, in our case the J48 and a priori algorithm; see Figures 2 and 3. The algorithms can either be applied directly to a dataset through a graphical user interface (known as GUI) or called from your own Java code [49].

## 3. Results and Discussion

We use the programming language GNU Octave for performing both univariate and multivariate statistical analysis [50]; we employ a 2-way contingency table analysis [31]. We also use the Weka software to perform our experiments with J48 and the a priori algorithm. We then feed the J48 and the a priori algorithms with the dataset shown in Table 2, that having 12 KIR genes along with the class variable (healthy and disease donors) for 343 patients (samples).

### 3.1. Statistical Analysis Results

The results of the univariate statistical analysis are in Table 5. The significant results (*p* < 0.05) are only for KIR2DL2 and KIR3DL1. There are neither motifs nor haplotypes associated to these two genes.

Traditional statistical comparison of KIR gene carrier frequencies (2 × 2 tables using Fishers' exact test) showed that KIR2DL2 was more frequent amongst the haematological malignancy cohort in comparison to the healthy individuals (77.8% versus 40.3%, resp.; *p* < 0.0001), Group A homozygosity was less frequent (11.1% versus 32%, resp.; *p* < 0.0044) and A,B heterozygous haplotypes were more frequent (86.7% versus 58.7%, resp.; *p* < 0.0002). This finding is interesting as KIR2DL2 is in tight linkage disequilibria (LD) with another gene, KIR2DS2. Both KIR2DL2 and KIR2DS2 are thought to bind HLA-C allotypes having C1 group specificity. Nevertheless, KIR2DL2 is an inhibitory protein whereas 2DS2 is activating. All genotyping reactions were carried out in triple, with further confirmatory runs if required. In addition, all genotyping was done at the same lab. As such, we are certain that this lack of LD is not related to technical issues. However, we cannot rule out that this might be the result of genotyping allele-dropout (failure to amplify a KIR2DS2 allele particularly common in the leukaemia cohort) or of cross-hybridization of 2DL2 oligonucleotides with other genes. This last possibility is unlikely as this genotyping approach has been previously validated and this finding does not occur in the healthy donor cohort.

For the multivariate statistical analysis, we take into account all 12 KIR genes variables. Therefore, we have $\sum_{i=2}^{12}\left(\begin{array}{c} 12\\ i \end{array}\right)=4083$ combinations. From these set of combinations, if we set our threshold to *p* ≤ 0.05 then we obtain 336 significant variable combinations. There are only 16 variable combinations associated to the haplotype cA01|tA01; see Table 6. If we set *p* ≤ 0.0001, then we obtain only 35 significant variable combinations and only one variable combination is associated to the haplotype cA01|tA01, that is, the variable combination #5 in Table 6. From the multivariate statistical analysis, the best variable combination is for KIR2DL1, KIR2DL2, KIR2DL3, KIR2DL5, KIR2DS4, KIR2DP1, and KIR3DL1; *p* value = 0.00002.

### 3.2. J48 Algorithm Results

In Figure 3, we show the results of the J48 algorithm. The only case when the donor is associated to a hematological malignancy (disease; *C* = 1) is when the gen KIR2DL2 is present (=1), KIR2DS2 is absent (=0), and KIR2DS4 is present (=1). There are not any motifs and haplotypes associated with this decision tree.

### 3.3. A Priori Algorithm Results

The a priori algorithm generates a total of 71,006 rules, taking in account only the rules where the class (*C*) appears at the consequent part of the rule, and there are only 12,052 rules associated to *C* = 1 (disease). In Table 7, we show only the first rules as generated by the a priori algorithm (where *C* = 1). The first 24 rules (out of 12,052) are more important because they are more frequent than the others. In Table 7, the frequency means that these rules are satisfied for 10 donors out of 43; that is, this pattern is present in 23% of the disease donors.

Because the variability in KIR genotype is such that most pairs of unrelated human individuals have different KIR genotypes, the unique feature of the human KIR system is the representation of two distinctive groups of haplotypes (A and B) [11]. Therefore, the more relevant rule given by the a priori algorithm, in Table 7, is the rule Id = 1870 (2DL1 = 1, 2DL2 = 1, 2DL3 = 1, 2DL5 = 1, 2DS2 = 0, 2DS4 = 1, 2DP1 = 1, 3DL1 = 1 ==> Class = 1). This rule refers to the haplotype cA01|tA01 [11], which is strongly inhibitory and then tolerates the tumors. In addition to this haplotype, two more inhibitory genes 2DL2 and 2DL5 are also present in this rule (which are part of the haplotype cB03), and the activating gen KIR2DS2 is absent. This association has been suggested for certain Hodgkin's lymphomas [38].

Moreover, it is clear, from Table 7, that the first 23 rules are a subset of the main rule (Id = 1870), the new discovered pattern. In fact, all of them have the same frequency. In other words, the first 23 rules are derivations from the rule #24 (Id = 1870); for example, the toy example shown above for the AND operator has the rule IF *g*1 = 0  ∧  *g*2 = 0 THEN *C* = 0, so the rules IF *g*1 = 0 THEN *C* = 0 and IF *g*2 = 0 THEN *C* = 0 are a subset of the previous rule. From Table 7, we can also infer that the genes KIR2DS1, KIR2DS3, KIR2DS5, and KIR3DS1 are somehow irrelevant, since they do not appear in any of these 24 rules.

Some researchers have reported some associations related to KIR2DS3 [35–37]. The rules shown in Table 7 (Class = 1) are only associated to disease (*C* = 1) with the absence of KIR2DS3. However, neither the J48 decision tree (Figure 3) nor the main rules generated by the a priori algorithm (Table 7) found some association between KIR2DS3 and disease.

### 3.4. Statistical Analysis versus the A Priori Algorithm

The unique feature of the human KIR system, which is not mirrored in other higher primates, is the representation of haplotypes (A and B). The haplotypes are present in all the >150 human populations studied [4]. Therefore, the association between haplotypes and disease is more important than only KIR genotype and disease.

In Table 8, we show the comparison between the multivariate statistical analysis and the a priori algorithm results. Table 8(a) shows the contingency table for the statistical analysis, and we can observe that this variable combination is associated to 18 disease donors (41%) of our study populations, although it is also associated to 46 healthy donors (15%). On the other hand, in Table 8(b), the contingency table for the rule found by the a priori algorithm shows that the rule is associated to 10 disease donors (23%), but it is not associated to any healthy donor. In other words, this rules is unique since it is only associated to disease donors. In fact, the *p* value and the *χ* ^2^ value show that the result is more statistically significant for the rule found by the a priori algorithm.

## 4. Conclusions

We studied a population of 300 healthy donors and 43 donors with haematological malignancies. The J48 algorithm and the univariate statistical analysis did not find any associations between haplotypes and disease. The multivariate analysis found 336 statistically significant variable combinations associated with the haplotype cA01|tA01 (*p* ≤ 0.05). From these set of combinations there is only one variable combination associated to this haplotype with *p* ≤ 0.0001 (see #5 in Table 6). This variable combination is associated to both disease and healthy donors (see Table 8). On the other hand, the a priori algorithm was able to discover a unique pattern through the rule Id = 1870. This pattern is more statistically significant than the variable combinations found by the multivariate statistical analysis (see Table 8). Moreover, the rule Id = 1870 is only associated to disease donors. In contrast, the variable combination found by the multivariate analysis is associated to both healthy and diseases donors. The rule Id = 1870 not only refers to the haplotype cA01|tA01, which is a predominantly inhibitory haplotype. This rule also refers to the genes KIR2DL2 and KIR2DL5, which are also inhibitory but not present in this haplotype which can be thought of more likely to tolerate tumours in our study population (with strict absence of KIR2DS2), that is, Mexican mestizos of San Luis Potosi State. This pattern was not discovered with previous studies on the same study population [33]. The methodology proposed in this paper provides a new insight into the analysis of datasets that allow researchers to find biomarkers for cancer and other diseases. Although the size and heterogeneity of our study cohort together with the lack of HLA typing data limits the clinical inferences that can be made from our results, it sets an example for a different way of analysing the clinical and functional relevance of complex genetic systems. Despite this, our methodology is able to discover patterns unseen for statistical analysis and decision trees generated by ID3 or J48 algorithms. The huge amount of rules generated by the a priori algorithm involves a data mining work to obtain the relevant rules. We found that the best performance is when a lower bound support is set to zero in combinations with a configuration that allows us to select rules only when the class is equal to one. The disadvantage of the a priori algorithm is that it requires huge computational resources (memory and processing). More research is needed to speed this algorithm up, and this may be the reason that this algorithm is not used in bioinformatics. A dataset with 23 variables is intractable for the Weka software with a personal computer. However, the dataset studied in this paper is able to run in the Weka software using a personal computer with a processor Intel Core i7 with 2.3 Ghz speed and 3 Gb memory. Undergoing investigations by our research group include the study of a dataset with KIR and HLA information of 413 HIV donors against our reference population of 300 healthy donors. We found that datasets with less 13 variables can be analysed on a personal computer regardless of the number of donors. Alternatively, there is commercial software to execute the a priori algorithm on a given dataset such as STATISTICA [51]; this software can manage more than 13 variables, but it also demands high computational resources.

## Figures and Tables

**Pseudocode 1:**
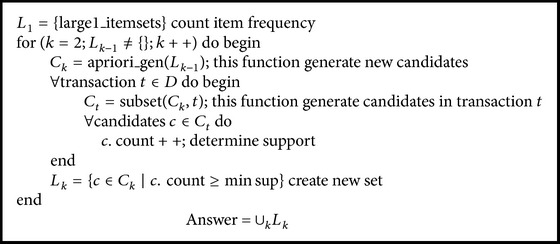


**Table 1:** Clinical data for the haematological cohort.

	*n*	%
Gender		
Male	23	53
Female	20	46

Diagnosis		
Chronic myeloid leukaemia	25	58
Hodgkin's lymphoma	18	42

B symptoms		
Present	30	70
Absent	13	30

ECOG^a^		
0	3	7
1	16	37
2	20	46
3	3	7
4	1	2

^a^Eastern Cooperative Oncology Group (ECOG).

**Table 2:** Study population; for visualization purposes, we only show the first five rows (disease, *C* = 1) and the last three rows (healthy, *C* = 0). Note that the last column corresponds to the class. Boxes with the mark *✓* indicate the presence of the gen (1), otherwise the absence (0).

Id	2DL1	2DL2	2DL3	2DL5	2DS1	2DS2	2DS3	2DS4	2DS5	2DP1	3DL1	3DS1	Disease (class—*C*)
1	✓	✓	✓			✓		✓		✓			1
2	✓	✓						✓		✓	✓		1
3	✓		✓					✓		✓	✓		1
4	✓		✓					✓		✓	✓		1
5	✓		✓	✓	✓			✓	✓	✓	✓	✓	1
⋮													⋮
341	✓		✓					✓		✓	✓		0
342	✓		✓					✓		✓	✓		0
343	✓	✓		✓	✓	✓			✓			✓	0

**Table 3:** Truth table, AND operator (∧).

*g*1	*g*2	*C* (class)
0	0	0
0	1	0
1	0	0
1	1	1

**Table 4:** This table contains 20 records; there are two variables (*g*1 and *g*2); the class *C* also represents 0 when the donor is healthy and 1 diseased.

#	*g*1	*g*2	*C* (Class)
1	1	1	1
2	0	0	0
3	0	1	0
4	1	1	1
5	0	0	0
6	1	0	0
7	0	1	0
8	1	1	1
9	0	0	0
10	0	1	0
11	1	1	1
12	0	0	0
13	1	0	0
14	0	1	0
15	0	1	0
16	1	1	1
17	1	1	1
18	0	0	0
19	0	1	0
20	0	0	0

**Table 5:** Univariate statistical analysis.

	2DL1	2DL2	2DL3	2DL5	2DS1	2DS2	2DS3	2DS4	2DS5	2DP1	3DL1	3DS1
*p* value	0.752	0.0000087	0.467	0.214	0.421	0.271	0.131	0.199	0.946	0.921	0.042	0.888
*χ* ^2^	0.100	19.764	0.530	1.547	0.649	1.213	2.281	1.649	0.005	0.010	4.128	0.020

**Table 6:** Multivariate statistical analysis; here we show only the variable combinations associated to the haplotype cA01|tA01. Boxes with the mark ✓ indicate that the variable is part of the variable combination; otherwise it is not taken in account.

#	2DL1	2DL2	2DL3	2DL5	2DS1	2DS2	2DS3	2DS4	2DS5	2DP1	3DL1	3DS1	*p* value	*χ* ^2^
1	✓	✓	✓					✓	✓		✓		0.00036	12.7
2	✓	✓	✓					✓		✓	✓	✓	0.01918	5.4
3	✓	✓	✓					✓	✓	✓	✓		0.00053	11.9
4	✓	✓	✓		✓			✓		✓	✓		0.00022	13.5
5	✓	✓	✓	✓				✓		✓	✓		**0.00002**	**17.4**
6	✓	✓	✓					✓	✓	✓	✓	✓	0.04289	4.09
7	✓	✓	✓		✓			✓		✓	✓	✓	0.01918	5.4
8	✓	✓	✓		✓			✓	✓	✓	✓		0.00574	7.6
9	✓	✓	✓	✓				✓		✓	✓	✓	0.01918	5.4
10	✓	✓	✓	✓				✓	✓	✓	✓		0.00213	9.4
11	✓	✓	✓	✓	✓			✓		✓	✓		0.00246	9.1
12	✓	✓	✓		✓			✓	✓	✓	✓	✓	0.04289	4.09
13	✓	✓	✓	✓				✓	✓	✓	✓	✓	0.04289	4.09
14	✓	✓	✓	✓	✓			✓		✓	✓	✓	0.01918	5.4
15	✓	✓	✓	✓	✓			✓	✓	✓	✓		0.00574	7.6
16	✓	✓	✓	✓	✓			✓	✓	✓	✓	✓	0.04289	4.09

**Table 7:** Rules generated by the a priori algorithm represented in tabular form. This figure contains only 24 rules with frequency 10, where the class = 1 (*C*).

#	Id	KIR2DL1	KIR2DL2	KIR2DL3	KIR2DL5	KIR2DS2	KIR2DS4	KIR2DP1	KIR3DL1	Frequency
1	1476		2DL2 = 1		2DL5 = 1	2DS2 = 0	2DS4 = 1			10
2	1477		2DL2 = 1		2DL5 = 1	2DS2 = 0			3DL1 = 1	10
3	1528	2DL1 = 1	2DL2 = 1		2DL5 = 1	2DS2 = 0	2DS4 = 1			10
4	1529	2DL1 = 1	2DL2 = 1		2DL5 = 1	2DS2 = 0			3DL1 = 1	10
5	1558		2DL2 = 1	2DL3 = 1	2DL5 = 1	2DS2 = 0	2DS4 = 1			10
6	1559		2DL2 = 1	2DL3 = 1	2DL5 = 1	2DS2 = 0			3DL1 = 1	10
7	1560		2DL2 = 1		2DL5 = 1	2DS2 = 0	2DS4 = 1	2DP1 = 1		10
8	1561		2DL2 = 1		2DL5 = 1	2DS2 = 0	2DS4 = 1		3DL1 = 1	10
9	1562		2DL2 = 1		2DL5 = 1	2DS2 = 0		2DP1 = 1	3DL1 = 1	10
10	1651	2DL1 = 1	2DL2 = 1	2DL3 = 1	2DL5 = 1	2DS2 = 0	2DS4 = 1			10
11	1652	2DL1 = 1	2DL2 = 1	2DL3 = 1	2DL5 = 1	2DS2 = 0			3DL1 = 1	10
12	1653	2DL1 = 1	2DL2 = 1		2DL5 = 1	2DS2 = 0	2DS4 = 1	2DP1 = 1		10
13	1654	2DL1 = 1	2DL2 = 1		2DL5 = 1	2DS2 = 0	2DS4 = 1		3DL1 = 1	10
14	1655	2DL1 = 1	2DL2 = 1		2DL5 = 1	2DS2 = 0		2DP1 = 1	3DL1 = 1	10
15	1681		2DL2 = 1	2DL3 = 1	2DL5 = 1	2DS2 = 0	2DS4 = 1	2DP1 = 1		10
16	1682		2DL2 = 1	2DL3 = 1	2DL5 = 1	2DS2 = 0	2DS4 = 1		3DL1 = 1	10
17	1683		2DL2 = 1	2DL3 = 1	2DL5 = 1	2DS2 = 0		2DP1 = 1	3DL1 = 1	10
18	1684		2DL2 = 1		2DL5 = 1	2DS2 = 0	2DS4 = 1	2DP1 = 1	3DL1 = 1	10
19	1784	2DL1 = 1	2DL2 = 1	2DL3 = 1	2DL5 = 1	2DS2 = 0	2DS4 = 1	2DP1 = 1		10
20	1785	2DL1 = 1	2DL2 = 1	2DL3 = 1	2DL5 = 1	2DS2 = 0	2DS4 = 1		3DL1 = 1	10
21	1786	2DL1 = 1	2DL2 = 1	2DL3 = 1	2DL5 = 1	2DS2 = 0		2DP1 = 1	3DL1 = 1	10
22	1787	2DL1 = 1	2DL2 = 1		2DL5 = 1	2DS2 = 0	2DS4 = 1	2DP1 = 1	3DL1 = 1	10
23	1806		2DL2 = 1	2DL3 = 1	2DL5 = 1	2DS2 = 0	2DS4 = 1	2DP1 = 1	3DL1 = 1	10
**24**	**1870**	**2DL1 = 1**	**2DL2 = 1**	**2DL3 = 1**	**2DL5 = 1**	**2DS2 = 0**	**2DS4 = 1**	**2DP1 = 1**	**3DL1 = 1**	**10**

**Table:** (a) Multivariate statistical analysis

	Disease	Healthy
Disease	18	25
Healthy	46	254

*p* value = 0.00002; *χ* ^2^ = 17.4.

**Table:** (b) A priori algorithm

	Disease	Healthy
Disease	10	33
Healthy	0	300

*p* value = 0.0; *χ* ^2^ = 71.86.
